# Gastrin exerts a protective effect against myocardial infarction via promoting angiogenesis

**DOI:** 10.1186/s10020-021-00352-w

**Published:** 2021-08-19

**Authors:** Jinjuan Fu, Yuanjuan Tang, Zhen Zhang, Lin Tong, Rongchuan Yue, Lin Cai

**Affiliations:** 1 Department of Cardiology, The Third People’s Hospital of Chengdu, Affiliated Hospital of Southwest Jiaotong University, College of Medicine, Southwest Jiaotong University, Chengdu, 610031 Sichuan People’s Republic of China; 2 College of Medicine, Southwest Jiaotong University, Chengdu, 610031 Sichuan People’s Republic of China; 3 Department of Cardiology, Affiliated Hospital of North Sichuan Medical College, Nanchong, 637000 Sichuan People’s Republic of China

**Keywords:** Gastrin, Myocardial infarction, Angiogenesis, Cardiac function, VEGF

## Abstract

**Background:**

It is known that increased gastrin concentration is negatively correlated with cardiovascular mortality, and plasma gastrin levels are increased in patients after myocardial infarction (MI). However, whether gastrin can play a protective role in MI remains unknown.

**Methods:**

Adult C57BL/6 mice were subjected to ligation of the left anterior descending coronary artery (LAD) and subcutaneous infusion of gastrin (120 μg/Kg body weight/day, 100 μL in the pump) for 28 days after MI. Plasma gastrin concentrations were measured through an ELISA detection kit. Mice were analyzed by echocardiography after surgery. CD31 and VEGF expression were quantified using immunofluorescence staining or/and western blot to assess the angiogenesis in peri-infarct myocardium. Capillary-like tube formation and cell migration assays were performed to detect gastrin-induced angiogenesis.

**Results:**

We found that gastrin administration significantly ameliorated MI-induced cardiac dysfunction and reduced fibrosis at 28 days in post-MI hearts. Additionally, gastrin treatment significantly decreased cardiomyocyte apoptosis and increased angiogenesis in the infarct border zone without influencing cardiomyocyte proliferation. In vitro results revealed that gastrin up-regulated the PI3K/Akt/vascular endothelial growth factor (VEGF) signaling pathway and promoted migration and tube formation of human coronary artery endothelial cells (HCAECs). Cholecystokinin 2 receptor (CCK_2_R) mediated the protective effect of gastrin since the CCK_2_R blocker CI988 attenuated the gastrin-mediated angiogenesis and cardiac function protection.

**Conclusion:**

Our data revealed that gastrin promoted angiogenesis and improved cardiac function in post-MI mice, highlighting its potential as a therapeutic target candidate.

## Background

Myocardial infarction (MI) is the leading cause of mortality for cardiovascular disease. Despite several therapeutic strategies that successfully restored the epicardial coronary flow, such as thrombolysis, surgical bypass grafting, and percutaneous coronary intervention (PCI), some patients remain ineligible for those therapies since microvasculature dysfunction promotes inefficient reperfusion of the entire myocardium (Laan et al. 2009). Hence, de novo formation of microvessel, also called angiogenesis, can rescue ischemic myocardium at the early stage after MI occurrence and is also essential for long-term cardiac remodeling to prevent the transition to heart failure (Cochain et al. 2013).

Gastrin is a peptide hormone secreted mainly by G-cells in the stomach after a meal (Varro and Ardill 2003). The physiological function of gastrin in regulating gastric acid secretion is currently well established. However, there is much more to discover concerning the biology of gastrin than is indicated. A study has shown that gastrin stimulated islet-cell growth and insulin secretion, which is proposed as a potential drug therapy for diabetes mellitus (Rehfeld 2019). Besides, gastrin plays an essential role in maintaining normal blood pressure (Chen et al. 2013) and protecting the kidney function against hypertensive injury (Gu et al. 2021). Diabetes and hypertension are important causes of coronary atherosclerotic heart disease. A previous study demonstrated that increased gastrin levels were negatively correlated with cardiovascular mortality risk (Goetze et al. 2016). Clinical data also revealed that serum gastrin levels were increased in patients with MI (Lapidus 1985; Tansey et al. 1981) and were associated with lower serum levels of cardiac troponin I in patients with unstable angina pectoris undergoing PCI (Yang et al. 2018). However, it is not clear if the increase in serum gastrin level is the result of MI or an attempt of the body to protect itself against cardiomyocyte damage after MI occurred. Additionally, gastrin is expressed in the heart and blood vessels (Gersl et al. 1981; Grossini et al. 2011). Two different studies reported that gastrin promoted a transient increase of Ca^2+^ (Grossini et al. 2013) and up-regulated nitric oxide production in porcine coronary artery endothelial cells (Grossini et al. 2012). Moreover, another study showed that gastrin increased coronary blood flow and myocardial contractility, dose-dependently, through intracoronary infusion in anesthetized pigs (Grossini et al. 2011). Besides, a work conducted by Yang et al*.* proposed that gastrin exerted a protective role against myocardial ischemia/reperfusion injury (IRI) in rats (Yang et al. 2018). Therefore, it is possible that gastrin may exert a protective effect against myocardial infarction.

An in vitro study revealed that gastrin induced chemotactic effects on endothelial cell migration and increased the tubulogenesis levels in endothelial cells, suggesting a potential pro-angiogenic role for this peptide (Clarke et al. 2006). Indeed, several studies found that gastrin induced pro-angiogenic effects in tumors (Bertrand et al. 2010; Lefranc et al. 2004). However, whether gastrin promotes cardiac angiogenesis after MI is still unclear. In this study, we aimed to investigate whether gastrin can improve the cardiac function after MI by increasing angiogenesis in the infarcted myocardium and to further explore its relative mechanism.

## Materials and methods

### MI mice model

C57/BL6 male mice (eight weeks old) were induced to myocardial infarction by the ligation of the left anterior descending (LAD) coronary artery as previously described (Li et al. 2020). Under isoflurane anesthesia, the LAD coronary arteries were ligated 2–3 mm far from the origin with a 7-0 silk suture. For the sham-operated control group, mice were exposed to all surgical procedures except the ligation of the LAD. Alzet osmotic minipumps (Model 1004, Durect Corporation, CA) were subcutaneously implanted in the mice dorsal region under isoflurane anesthesia. The minipumps were loaded with saline (control) or the chemicals and infused at a 2.64 μL/days rate for 28 days, according to the previous study (Huang et al. 2010). The infusate contained the saline vehicle (control, 2.64 μL/day, 100 μL in the pump), gastrin I (120 μg/Kg body weight/day, 100 μL in the pump, Cat. No. G1276, Sigma-Aldrich) (Gu et al. 2021; Rooman and Bouwens 2004), or/and the CCK_2_R inhibitor, CI988 (0.5 mg/Kg body weight/day, 100 μL in the pump, Cat. No. sc-205244A, Santa Cruz) (Xu et al. 1992). All procedures were approved by the Animal Use Subcommittee of Southwest Jiaotong University.

### Echocardiography

Echocardiography was performed using an echocardiographic monitor (Vivid9, GE Healthcare, WI), and 2% isoflurane was used to keep the mice under sedation. Hearts were observed in the short-axis with M-mode on the parasternal long. Diastolic left ventricle internal diameter (LVIDd) and systolic left ventricle internal diameter (LVIDs) were measured to find cardiac morphologic and functional changes. The percentage of fractional shortening (%FS) was calculated as (LVIDd– LVIDs)/LVIDd × 100. Also, the percentage of left ventricle ejection fraction (%EF) was calculated as ((LVIDd)^3^ -(LVIDs)^3^) (LVIDd)^3^ × 100. All of the echocardiography measurements were performed blindly.

### Plasma gastrin measurements

After an overnight fast, mice blood samples were collected from the orbital venous while under isoflurane anesthesia on the day of myocardial infarction (day 1), as well as on days 3, 5, 7, 9, 11, and 13. Blood samples were contained in EDTA-coated tubes then centrifuged at 1000 rpm for 5 min and plasma stored at −80 ℃ until assayed. Plasma gastrin concentration was determined by using a mice gastrin-17 ELISA detection kit (Cat. No. ml569213-J, mlbio, Shanghai) according to the manufacturer’s standard procedures.

### Histology

Mice heart tissues were fixed in 4% paraformaldehyde solution overnight at 25 °C and then undergoing paraffin embed and section. In order to analyze the myocardial fibrotic area after the myocardial infarction procedure with or without drug infusion, paraffin sections were cut through the entire ventricle from apex to base into serial sections with 0.5-mm intervals. After that, Masson’s trichrome staining (Cat. No. G1345, Solarbio, Beijing) was performed according to the manufacturer’s protocol following the dewaxing procedure.

### Apoptosis detection

An in situ apoptotic cell death detection kit based on the terminal deoxynucleotidyl transferase dUTP nick-end labeling (TUNEL, Cat. No. C1088, Beyotime, Shanghai, China) was used to detect apoptosis, according to the manufacturer’s standard procedures. Briefly, after the dewaxing procedure, cardiomyocytes were labeled with anti-cardiac troponin T antibody (cTnT, 1:100, Cat. No. MA5-12960, Invitrogen, MA) at 4 °C for 10 h (hrs), the nuclei were detected through 4’,6-diamidino-2-phenylindole (DAPI, Cat. No. C0065, Solarbio) staining. The TUNEL-positive cardiomyocytes were detected in 5 randomly selected nonadjacent images of peri-infarcted areas. The index of apoptosis was presented as the TUNEL-positive cardiomyocytes per 1 × 10^6^ Nuclei.

### Immunofluorescence staining

Paraffin-embedded hearts from all groups of mice were cut into 5 µm thick sections and then deparaffinized and dehydrated. After treatment with immunostaining blocking solution (Cat. No. P0102, Beyotime, Shanghai) for 1 h at room temperature, the sections were incubated with anti-cluster of differentiation 31 (CD31) primary antibody (1:100, Cat. No. GB11063-2, Servicebio), anti Ki67 antibody (1:100, Cat. No. 9129S, Cell Signaling Technology), and anti-cTnT antibody (1:100) for 10 h at 4 °C, subsequently washed three times with phosphate buffer saline and incubated with secondary antibodies conjugated with Alexa Fluor 488 or 555 (1:200, Cat. No. A-11001, A-21428, A-21422, Invitrogen, MA) for 2 h at 37 °C. DAPI was used for nuclear staining. The slides were mounted in an antifade mounting medium. Angiogenesis was detected by calculating the capillary density per field in the infarct border zone as previously reported. In order to quantify the number of Ki67^+^ (cell cycle marker) cardiomyocytes, the results were acquired from sections of the heart with at least five different fields and positions. In all cell-counting experiments, visual fields were randomized to reduce counting bias.

### Tube formation assay

Human coronary artery endothelial cells (HCAECs, Pricells, Wuhan, China) were cultured in primary endothelial cell basal medium (RPMI1640, Cat. No. A4192301, Gibco) supplemented with 10% fetal bovine serum (FBS, Cat. No.10099–141, Gibco), in a humidified incubator at 37 °C with 95% air/5% CO_2_ atmosphere as we previously described (Fu et al. 2016). All groups of cells were starved in a basal medium containing 1% FBS for 12 h before performing the experiments. The 24-well plate, pipette tips, and matrix gels were pre-cooled before tube formation assay. 300 μL Matrigel matrix (Cat. No. 354234, Corning, USA) was added to a 24-well plate and put into an incubator for gel formation. Subsequent to gel solidification, 500 μL of HCAECs suspension (2 × 10^5^/mL) was added to each well (Song et al. 2020). The plate was incubated at 37 °C. Tubule formation was observed using a Nikon inverted microscope (Tokyo, Japan) and determined by measuring branch number and length using the National Institutes of Health ImageJ software 6 h after treatment with gastrin (1 nM to 100 nM) or/and CI988 (100 nM). ImageJ software was used to analyze the tube lengths in 5 randomly selected visual fields.

### Cell migration assay

Transwell migration assay was performed as previously described. Briefly, cells were slowly put into the upper well of the transwell migration chamber (Cat. No. MCEP24H48, Millipore, Germany). After 24 h of treatment with gastrin (100 nM) or/and CY294002 (1000 nM, Cat. No. S1737, Beyotime, Shanghai, China) (Liu et al. 2016), the non-migrating cells were removed with a cotton swab, and the migrating cells on the underside of the membrane were stained with crystal violet. Cell migration was also analyzed using a scratch wound migration assay. Briefly, HCAECs were seeded into a 12-well plate in basal medium containing 10% FBS for 48 h. Culture media were then changed to basal medium containing 1% FBS for 12 h. Two parallel scratches were made by using a 10 µL pipette tip. Subsequently, HCAECs were treated with gastrin (100 nM) or/and CY294002 (1000 nM) for 8 h. The scratch was observed using a Nikon inverted microscope, as mentioned before. In order to calculating cell migration distance, HCAECs that migrated into the scratched area from the edge area were photographed and then measured in each image to yield an average value compared with the time zero point.

### Western blot

Protein was extracted from HCAECs after treatment with gastrin (1 nM to 100 nM), or/and CI988 (100 nM), or/and LY294002 (1000 nM) for 24 h. Western blotting was carried out as we previously described. Cells and heart tissue lysates containing 100 μg of protein were separated by SDS-PAGE and then electrophoretically transferred onto NC membranes (Bio-Rad, CA). After treatment with skimmed milk to blocking non-specific bands for 3 h, the blots were probed with the anti-phospho protein kinase B (Akt) antibody (1:800, Cat. No. 4060 T, Cell Signaling Technology), anti-Akt antibody (1:800, Cat. No. 9272S, Cell Signaling Technology), anti-phospho phosphatidylinositol 3 kinase (PI3K) antibody (1:800, Cat. No. 17366S, Cell Signaling Technology), anti-PI3K antibody (1:800, Cat. No. 4257S, Cell Signaling Technology), anti-VEGFA antibody (1:1000, Cat. No. 66828-1-Ig, Proteintech, Wuhan, China), anti-CD31 primary antibody (1:500, Cat. No.11265-1-AP, Proteintech), anti-Caspase 3 antibody (1:800, Cat. No. 19677-1-AP, Proteintech), anti-cleaved Caspase 3 antibody (1:500, Cat. No. 9661S, Cell Signaling Technology), and GAPDH (1:500, Cat. No. 60004-1-Ig, Proteintech) at 4 °C overnight. Membranes were washed three times with western washing buffer (TBST) and incubated with the corresponding secondary antibodies (Li-Cor, IRDye 800CW, 1:10000) for 2 h at room temperature. After that, NC membranes were washed three times using TBST again. Bound complexes were detected using the Odyssey Infrared Imaging System (Li-Cor Biosciences). Images were analyzed using the Odyssey Application Software to obtain the integrated intensities.

### Statistical analysis

Data were expressed as means ± standard deviation. The SPSS 20.0 software was used for general statistical analysis. Comparison within groups was made by repeated-measures ANOVA (or paired *t*-test when only two groups were compared), and the differences between groups were evaluated by factorial ANOVA with the Holm-Sidak post hoc test. Log-rank (Mantel-Cox) test was used for statistical survival analysis. A two-sided *P*-value less than 0.05 was considered statistically significant. Histograms were plotted using the GraphPad Prism 5.01 software.

## Results

### The plasma level of gastrin is increased in mice after MI

**Fig. 1:**
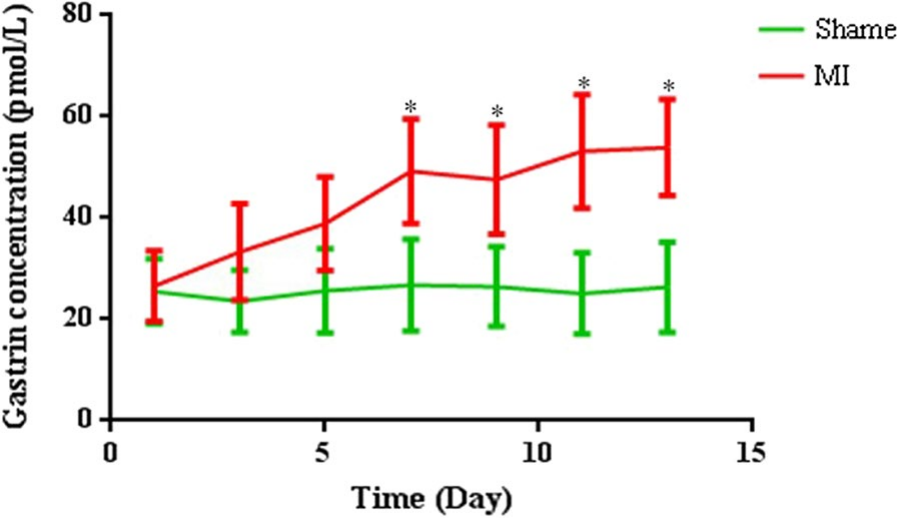
Comparison of plasma concentrations of gastrin at different days (day 1, 3, 5, 7, 11, and 13) in the myocardial infarction (MI) mice and in sham-operated mice. Data are expressed as the mean ± SD. n = 8. ^*^P < 0.05, vs. sham-operated mice

We induced MI by permanent ligation of the LAD in mice. To evaluate the changes in gastrin levels after MI, we detected the plasma concentration of gastrin in mice every two days for 13 days. Plasma concentration of gastrin was slightly increased in days 3 and 5, compared to the sham-operated group, in post-MI mice, but there were no significant differences. However, after 7 days, the plasma concentration of gastrin was significantly increased in the post-MI compared sham-operated group (Fig. 1). These data revealed that the plasma level of gastrin was increased in mice after MI.

### Gastrin improves cardiac function and survival rate in post-MI mice

**Fig. 2:**
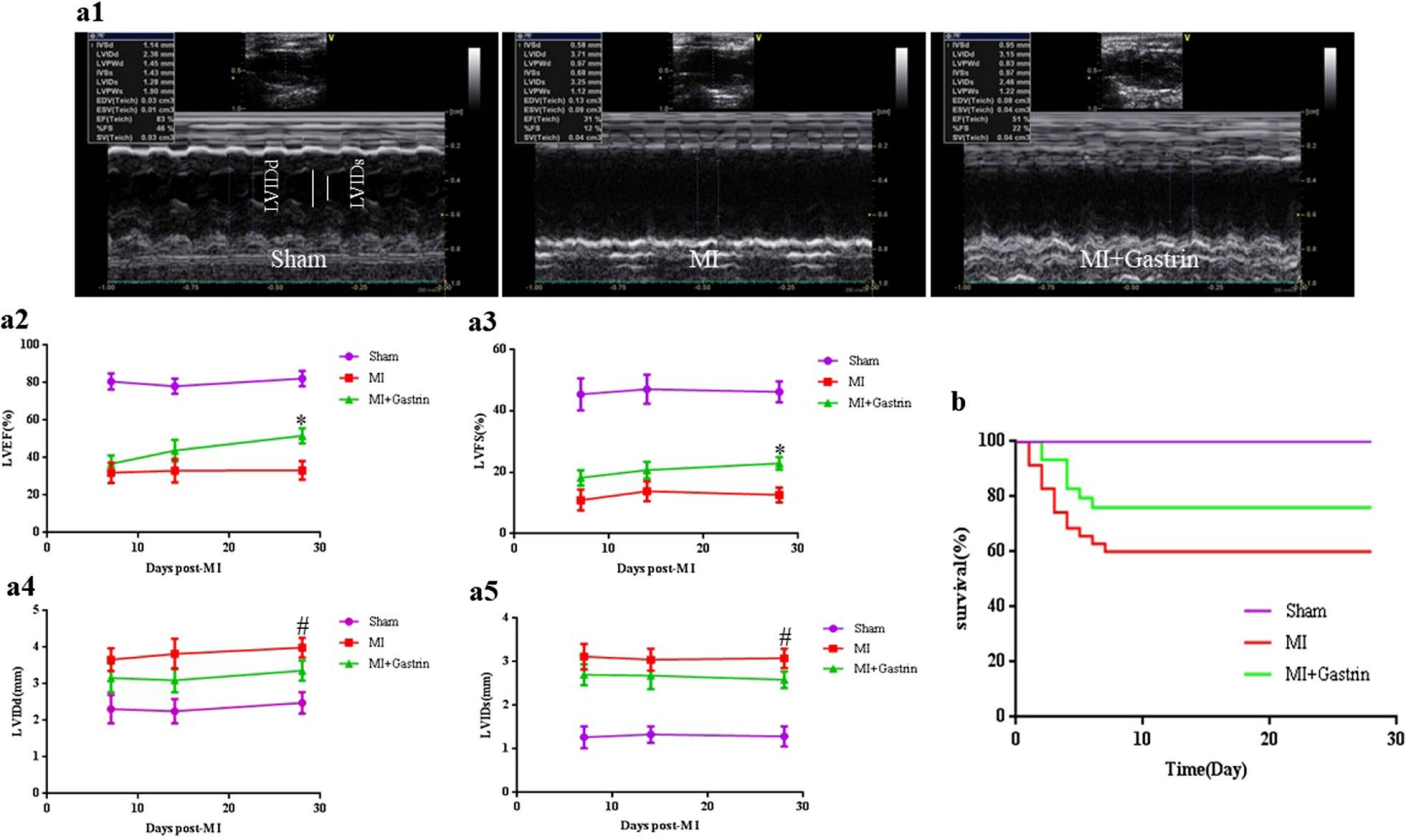
Gastrin ameliorates cardiac function and survival rate in myocardial infarction mice. **a1** Representative M-mode images of hearts with the sham operation or myocardial infarction 28 days after the saline vehicle and gastrin infusion. **a2–5** Represent left ventricle ejection fraction (EF), fractional shortening (FS), diastolic left ventricle internal diameter (LVIDd) and systolic left ventricle internal diameter (LVIDs) after MI. n = 10. ^*^P < 0.05, vs. MI mice; ^#^*P* < 0.05, vs. MI + Gastrin. **b** Gastrin improves Kaplan–Meier survival rate of mice subjected to MI. n = 10. Log-rank test *P* < 0.05

Ventricular function was evaluated in all groups to assess the gastrin effects on cardiac hemodynamics. After MI injury, mice were submitted to subcutaneous infusion of gastrin for 28 days. Cardiac function was measured by echocardiography at 7, 14, and 28 days after myocardial infarction (Fig. 2a1–a5). Our results suggested that compared with sham-operated mice, post-MI mice had increased ventricular dilation and decreased cardiac function. At 7 and 14 days, gastrin slightly increased LVEF and LVFS and slightly decreased LVIDd and LVIDs, but there were no statistical differences compared with the MI group. However, on day 28 post-MI, gastrin improved LVEF and LVFS compared with the control MI mice. Besides, gastrin-treated mice had statistically smaller left ventricle size than MI mice, as indicated by the significant reduction of LVIDd and LVIDs. Meanwhile, survival rates of gastrin-treated mice were lower than the control mice subjected to MI (Fig. 2b). These data demonstrated that gastrin improved cardiac function in post-MI hearts.

### Gastrin decreases cardiomyocyte fibrosis and apoptosis in post-MI hearts

**Fig. 3:**
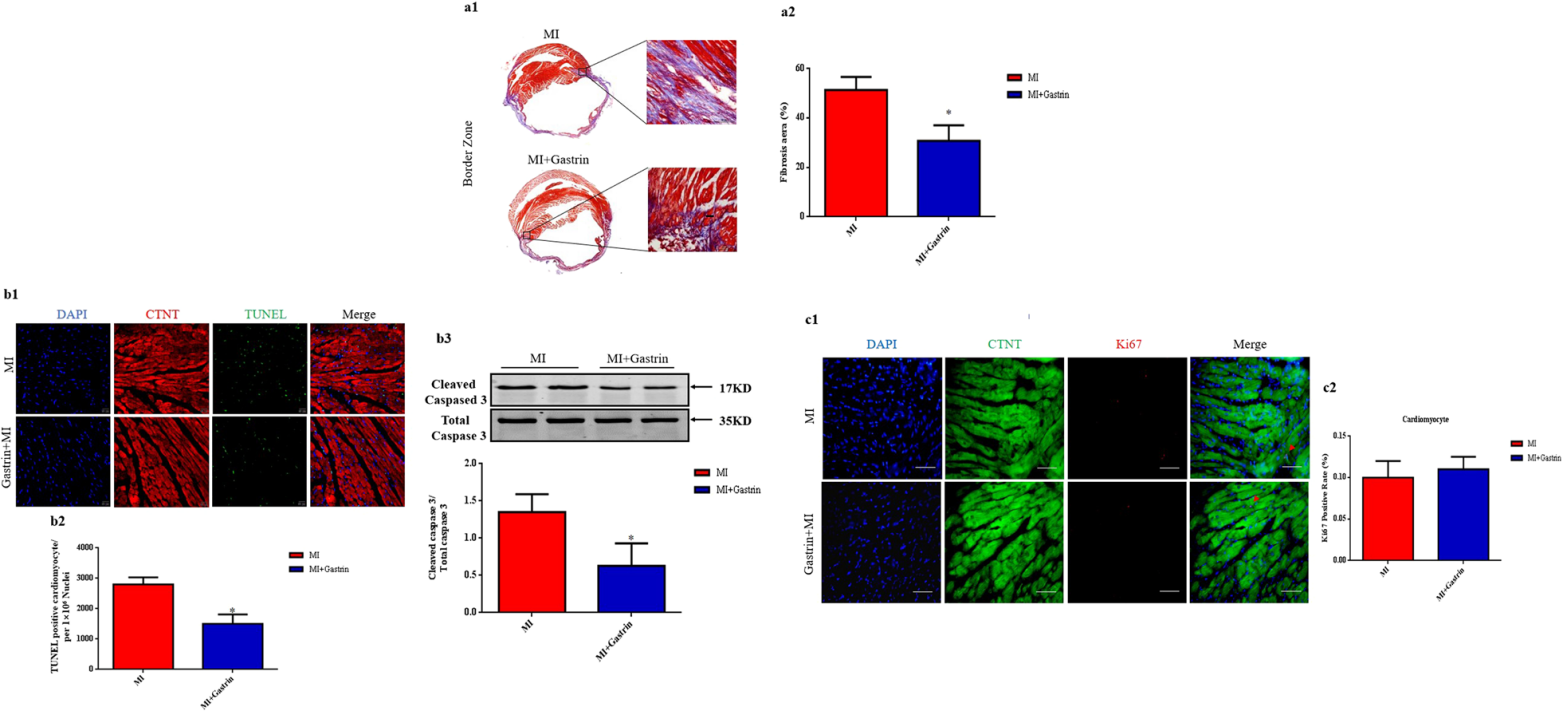
Gastrin decreases cardiomyocyte apoptosis and fibrosis after myocardial infarction. **a** Representative images (**a1**) and quantification (**a2**) of the fibrotic area in infarct border zone by Masson trichrome staining at 28 days post-MI. **b** Representative images (**b1**) and quantification (**b2**) of terminal deoxynucleotidyl transferase dUTP nick-end labeling–positive (TUNEL^+^) cardiomyocytes in infarct border zone at 2 days after MI. TUNEL^+^ nuclei are stained green, cardiac troponin T antibody (cTnT) is red, and 4'-6-diamidino-2-phenylindole (DAPI) is blue. **b3** Western blot was used to measure the protein levels of Caspase 3 in the infarct border zone at 2 days after MI. Results are expressed as the ratio of cleaved caspased 3 to total caspase 3. **c**, Representative images (**c1**) and quantification (**c2**) of cardiomyocyte proliferation 28 days after myocardial infarction, through Ki67 immunostaining. Cardiac troponin T antibody (cTnT) is green, Ki67 is red, and 4'-6-diamidino-2-phenylindole (DAPI) is blue. **a** and **c** Scale bar = 50 µm. **b** Scale bar = 20 µm. n = 8. ^*^P < 0.05, vs. MI mice. *P* = NS means no significant difference

Masson’s trichrome staining was performed to evaluated fibrosis 4 weeks post-MI. The result indicated that gastrin-treated mice had decreased fibrosis level in the peri-infarct border zone compared with the control MI group (Fig. 3a1–a3). Cardiomyocytes are susceptible to apoptosis during MI (Matsumoto et al. 2019). Gastrin has been reported to protect cells from apoptosis (Ramamoorthy et al. 2004) and promote cell proliferation (Duckworth et al. 2013). Next, we evaluated cardiomyocyte apoptosis and proliferation levels in the hearts. Apoptosis was detected in the peri-infarct border zone collected two days post-MI with TUNEL staining (Li et al. 2020). Cardiomyocytes were identified by cTnT staining. We found that the gastrin-treated group had decreased TUNEL-positive cardiomyocytes (Fig. 3b1–b2). Moreover, western blot data revealed that the expression of cleaved caspase-3 was also significantly decreased in gastrin-treated MI mice than control MI mice. These data suggested that gastrin protected the myocardium partially by reducing cardiomyocyte apoptosis. Cardiomyocyte proliferation was determined by Ki67 staining. Our study found that the rate of Ki67 positive cardiomyocytes was comparable between the gastrin-treated MI mice and the control mice (Fig. 3c1–c2), indicating that gastrin does not influence cardiomyocyte proliferation ability in the post-MI heart.

### Gastrin promotes angiogenesis after myocardial infarction

**Fig. 4:**
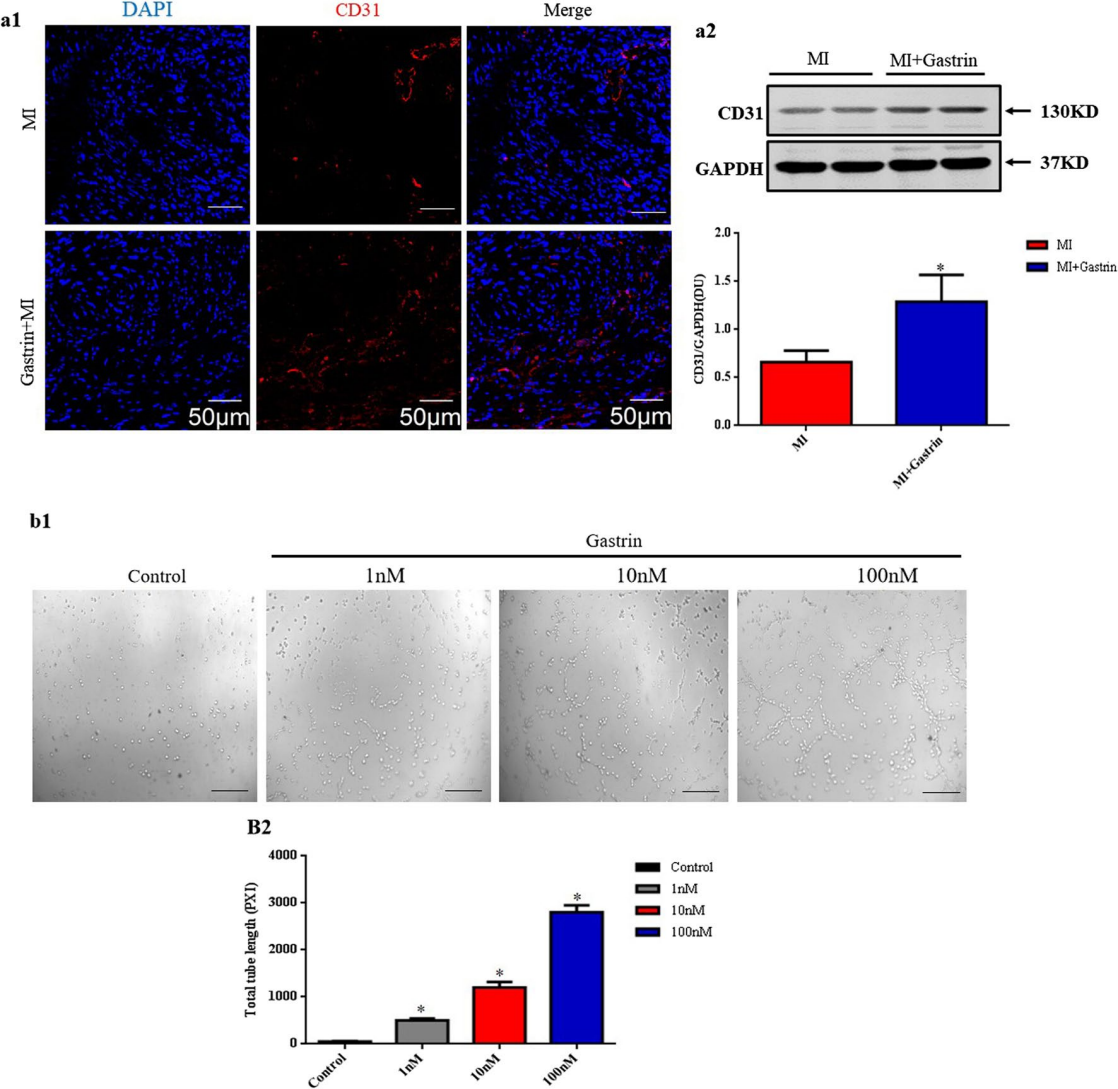
Gastrin induces angiogenesis after myocardial infarction. **a1** Immunofluorescence staining with anti-cluster of differentiation 31 (CD31) antibody showed the microvessel densities in the infarcted border zone 14 days after MI. DAPI is blue, and CD31 is red. Scale bar = 50 µm. **a2** Western blot was used to measure the protein levels of CD31 in the infarcted border zone of the heart 14 days after MI. Results are expressed as the ratio of CD31 to GAPDH. n = 8. ^*^P < 0.05, vs. MI mice. **b** Human coronary artery endothelial cells (HCAECs) were incubated with gastrin at the indicated concentrations. Representative images (**b1**) and quantification (**b2**) of tube formation assay with HCAECs. Scale bar = 50 µm. n = 4. ^*^P < 0.05, vs. Control

Previous studies have shown that gastrin induced chemotactic effects on endothelial cell migration and increased the tubulogenesis levels of the endothelial cells (Clarke et al. 2006). Insufficient post-MI angiogenesis has been regarded as a non-negligible event that promotes heart failure development. In contrast, stimulating angiogenesis ameliorates cardiac remodeling after myocardial infarction (Laan et al. 2009). CD31 plays an important role in endothelial cell intercellular junctions and is widely used as a marker for angiogenesis quantification (Feng et al. 2020; Ho et al. 2012). We, therefore, assessed the de novo formation of microvessel in peri-infarct myocardium at 14 days post-MI through CD31 staining and expression. Both immunofluorescence staining and Western blot showed that CD31 expression in the peri-infarct border zone were increased in gastrin-treated mice (Fig. 4a1–a2). The in vitro experiment also revealed that gastrin (1 nM–100 nM) promoted tube formation of HCAECs in a concentration-dependent manner (Fig. 4b1–b2). These results suggested that gastrin improves angiogenesis in the post-MI heart.

### Gastrin induces angiogenesis via the PI3K/Akt/VEGF signaling pathway

**Fig. 5:**
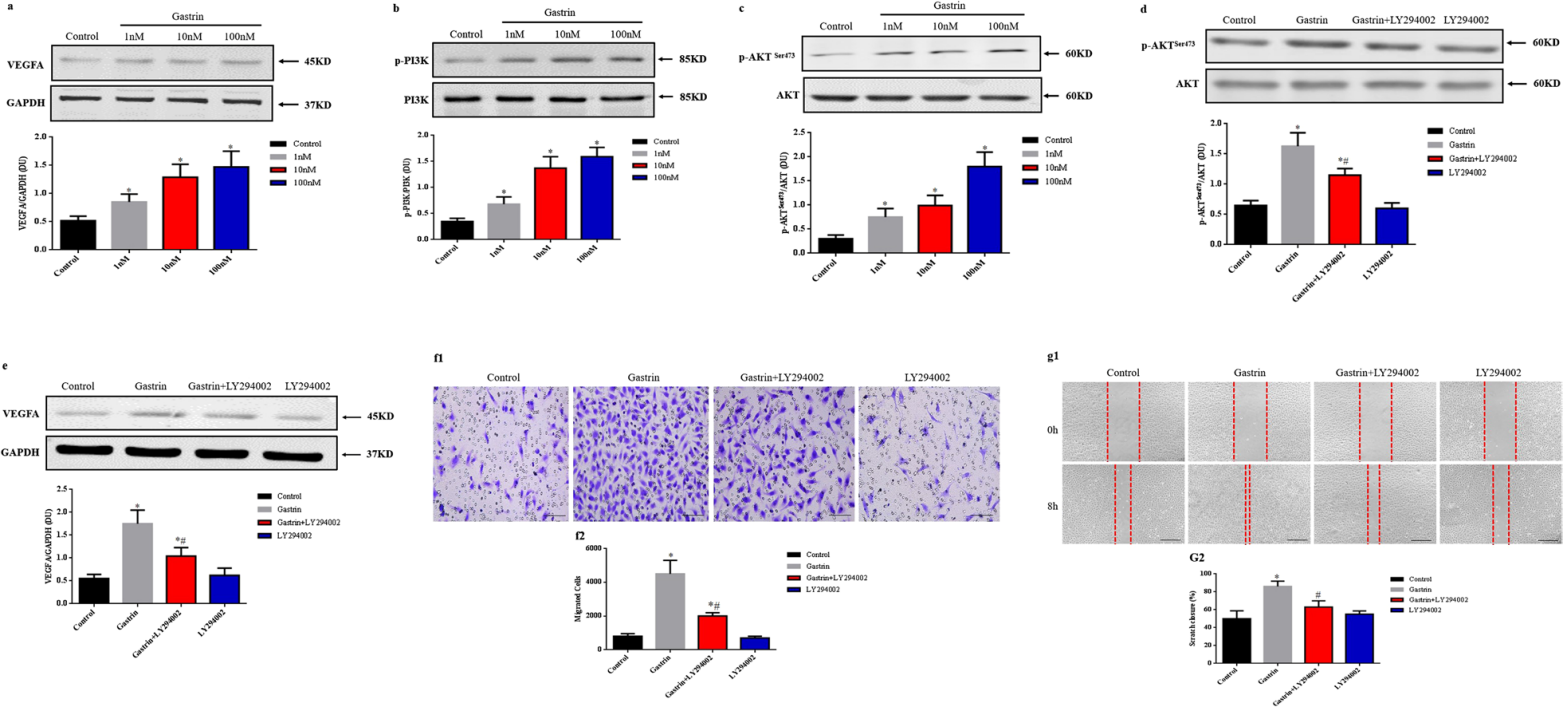
PI3K/Akt/VEGF signaling pathway is involved in the pro-angiogenic effect of gastrin. Endothelial cells were incubated with gastrin at the indicated concentrations for 24 h. VEGFA (**a**), phospho-PI3K (**b**), and phospho-Akt (**c**) results were normalized by GAPDH, total PI3K, and Akt, respectively. Endothelial cells were treated with gastrin (100 nM, 24 h) in the presence or absence of a PI3K inhibitor, LY294003 (1000 nM). Phospho-Akt (**d**) and VEGFA (**e**) were normalized by GAPDH and total Akt, respectively. n = 4. ^*^P < 0.05, vs. Control group; ^#^*P* < 0.05, vs gastrin-treated group. **f** Representative images and quantification of endothelial cell migration detected by transwell migration assay. **g**, Representative images and quantification of endothelial cell migration detected by scratch wound-healing assay. **f** and **g** scale bar = 50 µm. n = 4. ^*^P < 0.05, vs. Control group; ^#^*P* < 0.05, vs. gastrin-treated group. VEGF, vascular endothelial growth factor; PI3K, phosphatidylinositol 3 kinase; Akt, protein kinase B; DU, density units; p, phospho

Vascular endothelial growth factor (VEGF), a key factor known to stimulate angiogenesis, attenuates cardiac dysfunction after MI in animal models (Cui et al. 2018; Rasanen et al. 2021). Previous studies reported that gastrin induced angiogenesis via stimulating VEGF signaling pathway (Bertrand et al. 2010). Liu et al*.* found that gastrin attenuated kidney ischemia/reperfusion injury through PI3K/Akt mediated anti-apoptosis signaling pathway (Liu et al. 2020). PI3K/Akt signaling also plays a critical role in angiogenesis (Liang et al. 2019; Sun et al. 2014). Therefore, we measured the phosphorylation status of PI3K, Akt, and VEGFA protein levels. As shown in Fig. 5a–c, after 24 h of treatment, gastrin (1 nM–100 nM) increased PI3K and Akt (Ser^473^) phosphorylation and VEGFA expression in a concentration-dependent manner in HCAECs but had no effect on total PI3K and Akt. Besides, gastrin (100 nM) treatment increased Akt phosphorylation and VEGFA protein level, which can be blocked by PI3K inhibitor, LY294002 (Fig. 5d–e). Endothelial cell migration is essential for angiogenesis. In our transwell migration study, gastrin (100 nM) treatment significantly boosted HCAECs migration, which was partially inhibited by LY294002 (Fig. 5f1–f2). Besides, the scratch wound healing result also revealed that HCAECs migration was statistically increased by gastrin (100 nM), which was partially repressed by LY294002 (Fig. 5g1–g2). These results suggested that the gastrin-induced angiogenesis, at least partly, was dependent on PI3K/Akt/VEGF signal pathway.

### Gastrin, via CCK_2_R, exerts a protective role against myocardial infarction

**Fig. 6:**
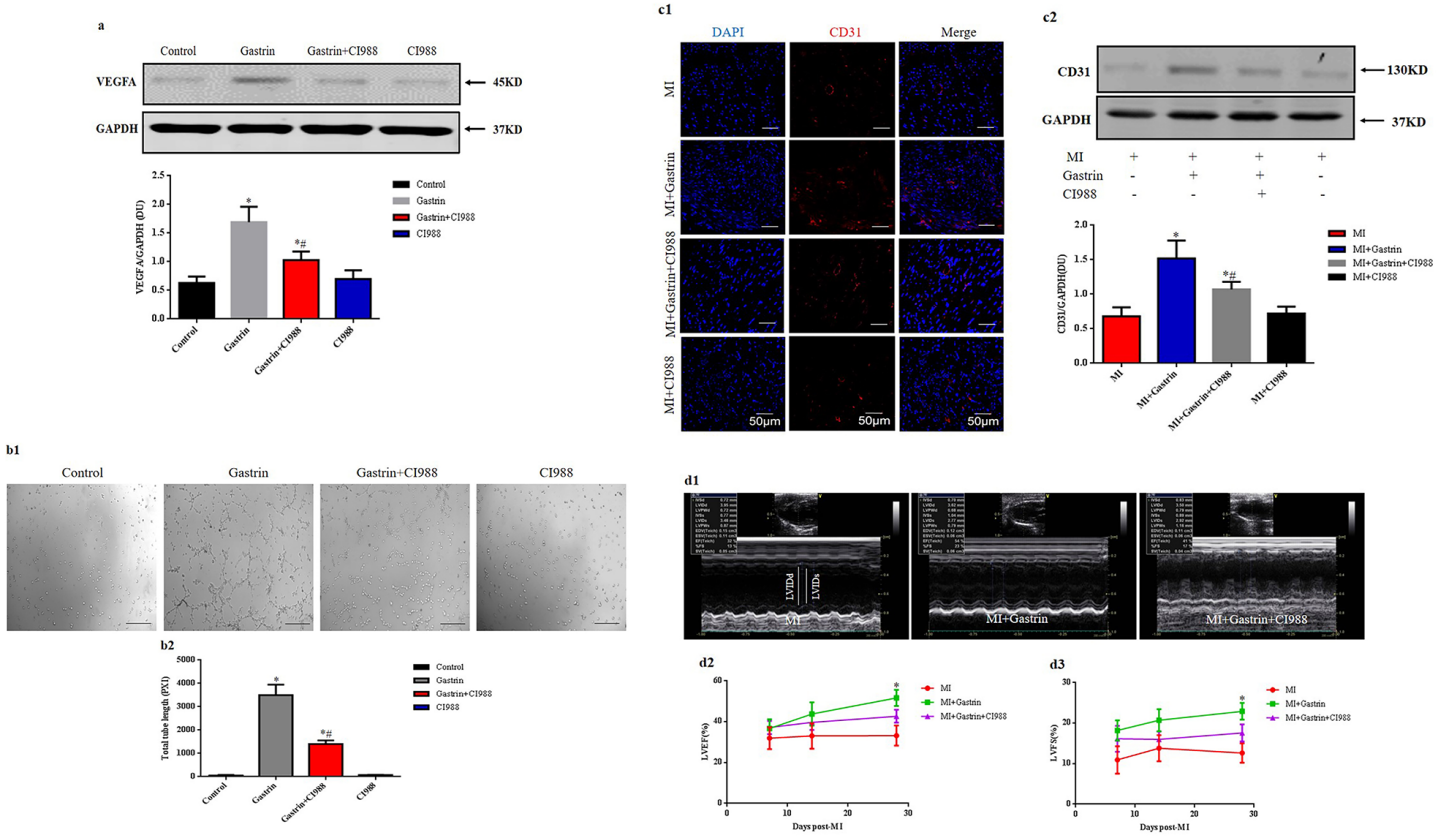
CCK_2_R role in the protective effects of gastrin against myocardial infarction. Endothelial cells were treated with gastrin (100 nM) with or without the CCK_2_R inhibitor, CI988 (100 nM). **a** Western blot was used to detect the protein levels of vascular endothelial growth factor (VEGFA). Results are expressed as the ratio of VEGFA to GAPDH. n = 4. ^*^P < 0.05, vs. Control; ^#^*P* < 0.05, vs. gastrin-treated group. **b** Representative images and quantification of endothelial cell tube formation assay in vitro. Scale bar = 50 µm. n = 4. ^*^P < 0.05, vs. Control; ^#^*P* < 0.05, vs gastrin-treated group. Myocardial infarction mice were infused with gastrin or/and CI988. **c1** Immunofluorescence staining with anti-cluster of differentiation 31 (CD31) antibody showed the microvessel densities in the infarcted border zone 14 days after MI. DAPI is blue, and CD31 is red. Scale bar = 50 µm. **c2** Western blot was used to detect the protein levels of CD31 in the peri-infarct area of the heart 14 days after MI. Results are expressed as the ratio of CD31 to GAPDH. n = 8. ^*^P < 0.05, vs. MI group; ^#^*P* < 0.05, vs gastrin-treated MI mice. **d1** Representative M-mode images of hearts with MI 28 days after gastrin or/and CI988 infusion. **d2–3** Left ventricle ejection fraction (EF), fractional shortening (FS) 7, 14, and 28 days after MI. n = 8. ^*^P < 0.05, vs. MI + Gastrin + CI988

Gastrin and cholecystokinin (CCK) share the same receptor (Guilloteau et al. 2006). Cholecystokinin receptor (CCKR) has 2 subtypes, type1 and type 2. The expression of CCK_2_R is much more than CCK_1_R and has higher affinity for gastrin than CCK_1_R in the coronary arteries (Dockray et al. 2005). In the in vitro study, we found that the gastrin-mediated upregulation of VEGFA was blocked by CI988 (100 nM), a specific CCK_2_R inhibitor (Fig. 6a). The pro-angiogenic effect of gastrin was also inhibited in the presence of CI988 (Fig. 6b). Moreover, gastrin up-regulated CD31 expression in the border zone (Fig. 6c1–c2), and the improved cardiac function (Fig. 6d1–d2) was also abolished in the presence of CI988. These data indicated that CCK_2_R mediated the protective role of gastrin against MI.

## Discussion

Studies on several gastrointestinal hormones have demonstrated that there may exist a gastro-heart axis that protects cardiovascular function. For instance, glucagon-like peptide 1 (GLP-1) has been reported to protect myocardial ischemia/reperfusion injury (Andrikou et al. 2019; Bonaventura et al. 2019). A study also found that ghrelin may be essential in ameliorating the efficacy of mesenchymal stem cell-based therapy for ischemic heart disease (Han et al. 2015). Besides, cholecystokinin was proposed to improve cardiac function in endotoxic shock rats (Saia et al. 2013). Gastrin, as one of the main gastrointestinal hormones, together with cholecystokinin, was among the first gastrointestinal hormones discovered. The two peptides were structurally related and activated the same receptor (the CCK_2_ receptor) (Zeng et al. 2020). Serum gastrin level markedly increased after a meal, about 20-fold more than cholecystokinin (Rehfeld et al. 2007). A previous study reported that the serum concentration of gastrin was significantly increased after myocardial infarction (Tansey et al. 1981). Besides, high serum gastrin level was reported to be an independent predictor of MI (Lapidus 1985). Similarly, our result also revealed that the plasma level of gastrin was increased in mice after MI. However, the pathophysiological significance of this phenomenon remains to be elucidated.

A former study found that increased gastrin concentration might represent an adaptive response to hypoxic conditions (Laval et al. 2015). Indeed, Yang et al*.* proposed that gastrin protects against myocardial IRI through activation of RISK (Reperfusion Injury Salvage Kinase) and SAFE (Survivor Activating Factor Enhancement) pathways (Yang et al. 2018). Also, gastrin was reported to protect the brain from ischemia-induced dysfunction in stroke-prone spontaneously hypertensive rats (Yasui and Kawasaki 1995). More recently, gastrin attenuated renal IRI through anti-apoptosis signaling (Liu et al. 2020). Our data showed that gastrin treatment for 28 days improved cardiac function in the post-MI hearts. Earlier works have shown that gastrin displays proliferative and antiapoptotic effects (Duckworth et al. 2013; Ramamoorthy et al. 2004). Our data revealed that gastrin ameliorated apoptosis but did not increase cardiomyocyte proliferation in the post-MI heart.

Pro-angiogenic therapy has the potential to rescue the ischemic myocardium at the early stages after MI and is also essential for long-term left ventricular remodeling to prevent the transition to heart failure (Li et al. 2021). However, angiogenesis after myocardial infarction is insufficient. Thus, stimulating angiogenesis is an essential strategy for MI patients. Angiogenesis is based on endothelial cell proliferation and migration to form new vessels through sprouting in the hypoxic or ischemic region (Carmeliet and Jain 2011). Our in vivo study revealed that CD31-positive microvessels in the infarct border zone were increased by gastrin treatment after LAD ligation for 14 days, and the in vitro assay showed that migration of HCAECs and tube formation were stimulated by gastrin, meaning that gastrin promoted angiogenesis in ischemic myocardium.

VEGF, a critical growth factor for vascular endothelial cells, is a critical factor that regulates angiogenesis and attenuates cardiac dysfunction after MI in animal models (Carmeliet and Jain 2011; Saif and Emanueli 2014). Although there are other related genes, including VEGF-B and VEGF-C, VEGF-A plays a dominant role in regulating angiogenesis (Apte et al. 2019). PI3K/Akt signaling plays an important role in angiogenesis and exerts a protective role in myocardial infarction (Peng et al. 2021; Ruan et al. 2020). Bertrand et al*.* found that gastrin-gly stimulated VEGF expression through the PI3K/Akt pathway (Bertrand et al. 2010). In porcine coronary artery endothelial cells, gastrin has been reported to stimulate NO generation through PI3K/Akt signaling pathway (Grossini et al. 2012). We, therefore, performed a western-blot experiment using lysates from HCAECs and found that gastrin increased PI3K and Akt phosphorylation and VEGFA expression in a concentration-dependent manner, and this process was partly repressed by PI3K inhibitor LY294002. Similarly, the increased HCAECs migration stimulated by gastrin was also partly blocked by LY294002. These results indicated that the PI3K/Akt/VEGF signal pathway was involved in the pro-angiogenic effect of gastrin in post-MI mice.

The gastrin peptide family has two subtype receptors, namely, CCK_1_R and CCK_2_R. The expression of CCK_2_R is greater than CCK_1_R in the coronary arteries and has a higher affinity for gastrin than CCK_1_R (Grossini et al. 2013). Gastrin has been reported to up-regulate NO production in the artery, and the intracoronary infusion of gastrin significantly increased coronary blood flow through stimulating CCK_2_R in pigs (Grossini et al. 2011). In this study, we showed that CCK_2_R inhibitor C1988 abolished the pro-angiogenic function of gastrin. Therefore, the potential cardioprotective role of gastrin in post-MI was probably mediated by the CCK_2_R.

## Conclusion

We demonstrated that increased plasma gastrin level played a protective role against cardiac dysfunction after myocardial infarction. Gastrin protected the post-MI heart from dysfunction by reducing cardiomyocyte apoptosis and promoting angiogenesis. The pro-angiogenic effect of gastrin was associated with CCK_2_R mediated PI3K/Akt/VEGF signal pathway activation.
