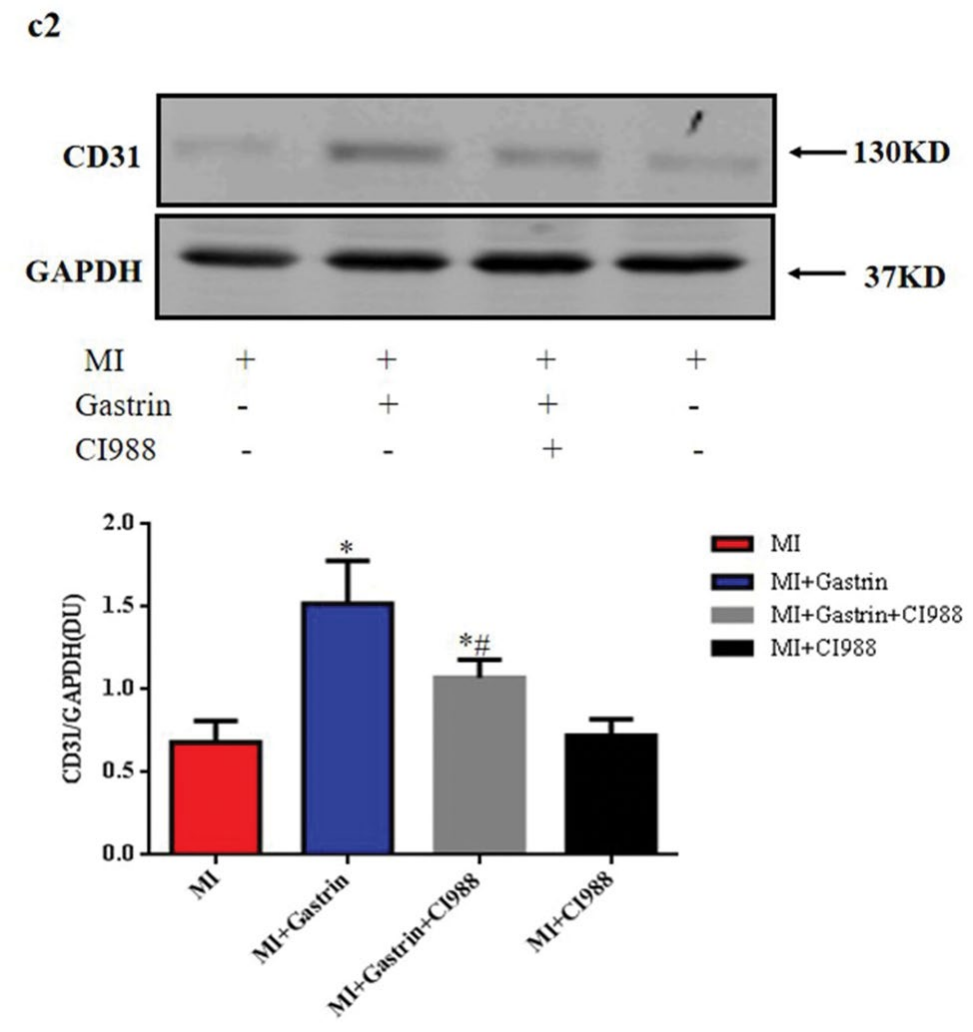# Correction: Gastrin exerts a protective effect against myocardial infarction via promoting angiogenesis

**DOI:** 10.1186/s10020-025-01256-9

**Published:** 2025-05-29

**Authors:** Jinjuan Fu, Yuanjuan Tang, Zhen Zhang, Lin Tong, Rongchuan Yue, Lin Cai

**Affiliations:** 1 Department of Cardiology, The Third People’s Hospital of Chengdu, Affiliated Hospital of Southwest Jiaotong University, College of Medicine, Southwest Jiaotong University, Chengdu, 610031 Sichuan People’s Republic of China; 2 College of Medicine, Southwest Jiaotong University, Chengdu, 610031 Sichuan People’s Republic of China; 3 Department of Cardiology, Affiliated Hospital of North Sichuan Medical College, Nanchong, 637000 Sichuan People’s Republic of China

**Correction: Molecular Medicine (2021) 27:90**

10.1186/s10020-021-00352-w

In this article (Fu et al. 2021) the wrong figure appeared as Fig. 6 (c2); the figure should have appeared as shown below.

The original article has been corrected.

Incorrect Fig. 6 (c2).

**Figure:**
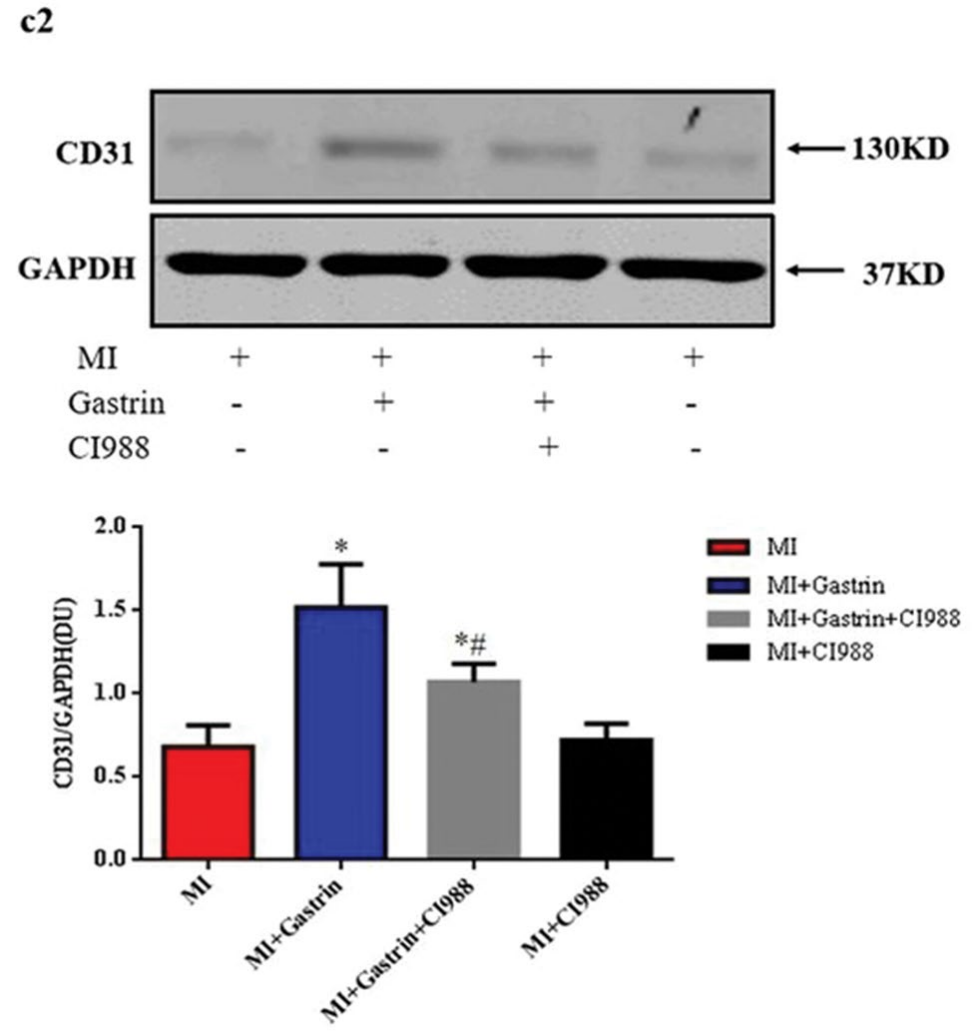

Correct Fig. 6 (c2).

**Figure:**